# Surgical Implications of Asymmetric Distribution of Dermal Collagen and Elastic Fibres in Two Orientations of Skin Samples from Extremities

**DOI:** 10.1155/2014/364573

**Published:** 2014-12-23

**Authors:** Naveen Kumar, Pramod Kumar, Satheesha Nayak Badagabettu, Keerthana Prasad, Ranjini Kudva, Raghuveer Coimbatore Vasudevarao

**Affiliations:** ^1^Department of Anatomy, Melaka Manipal Medical College, Manipal Campus, Manipal University, Manipal 576104, India; ^2^Department of Plastic Surgery, King Abdul Aziz Hospital, Sakaka, Al-Jouf 42421, Saudi Arabia; ^3^Department of Information Science, Manipal School of Information Science, Manipal University, Manipal 576104, India; ^4^Department of Pathology, Kasturba Medical College, Manipal University, Manipal 576104, India; ^5^Department of Pathology, Yenepoya University, Deralakatte, Mangalore 575018, India

## Abstract

*Background.* Clinically, scar related complications are observed to be dissimilar in different regions of the body. Unequal distribution of dermal collagen and elastic fibres in different orientations could be one of the multifocal causes of scar related complications, for which this evaluating study has been taken up. *Materials and Method.* 300 skin samples collected in horizontal and vertical orientations were studied histomorphometrically. This study involved image analysis of specially stained histological section using tissue-quant software. The outcome result was termed as quantitative fraction. From the result, various ratio values were also calculated for the ratio analysis. *Results.* The differences in the quantitative fraction of dermal elastic content between 2 directions were statistically significant at joint areas (shoulder joint, wrist, and ankle) (*P* < 0.001) but for collagen, significant difference was observed at shoulder joint and wrist only. Dermis of the forearm and thigh did not show any differences in their collagen content, but for elastic, thigh did show a significant difference while forearm had no change between 2 directions. *Conclusion*. Analysis of unequal content of dermal element in two directions under the perspective of wound healing consequences is subjective depending upon the anatomical position and functional status of the areas.

## 1. Introduction

Collagen and elastic fibres are the two major dermal connective tissue populations that exhibit their functional significance during wound healing process. Various studies in this aspect have emphasized their complimentary functional attribution in the process of minimizing the scar formation [1]. The scar tissue resulting from the process of wound healing also has similar types of collagen as in normal skin but with a deviated pattern of arrangement and distribution from the normal [2].

For many years, the well-known direction followed to make incisions to obtain aesthetic scar has been Langer's line or cleavage line. There have been many other concepts of lines on the skin which have been put forward and made the concept of Langer's line debatable. Borges studied intensely the lines of skin tension and comprehended with seven best known skin tension lines. According to him, single best choice of line is still questionable to fulfill complete satisfaction of wound healing [3]. Most popular among these is the Langer's line, concept of which lies on the basis of pattern of arrangement of dermal collagen in a particular direction. Previous works on the quantification of dermal collagen and elastic fibres in two different directions of skin samples obtained from the same area of the human body did confirm the asymmetric distribution of them [1, 4, 5]. The results of these studies attempted a hypothetical explanation which possibly explains pleomorphic behaviour of scar in different parts of the body.

Plastic surgeons on the other hand, in their personal experience and observations in the clinical setup, are still in dilemma about the speckled behaviour of scar, even after the incisions are made on the skin according to the standard lines of choice. This made them theorize the possible role of varied quantity and content of dermal collagen together with elastic fibres in different orientations of skin plane in addition to their normal coalition pattern in the dermis. For research purpose, quantification of dermal connective tissue fibres using image analysis technique was reported to be more relevant than the observer's ratings as it is accurate and comparable to polarized light technique [6]. The percentage area occupied by the tissue structures demonstrated by biological stains can be measured by image analysis and its accuracy and reliability have been proved by previous studies [1, 4, 5, 7]. Using similar methodology, histomorphometric analysis of dermal collagen and elastic fibres in two directions from the areas of head and neck [4] and trunk region [5] confirmed its asymmetric distribution as well as diverged content among themselves whose significance was in justification with the observation of life.

## 2. Materials and Methods

**Figure 1:**
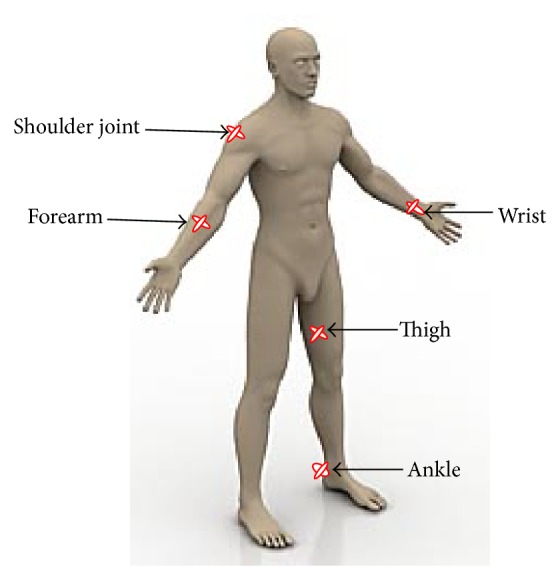
Topographic areas of sample collection from extremity region of human body (adapted from http://www.designyourway.net/blog/).

Current study involved 300 skin samples collected in two orientations (horizontal and vertical) from five areas (shoulder joint, wrist, ankle, forearm, and thigh) of extremity region of human cadaver. The skin samples were obtained from 30 formalin embalmed healthy looking human cadavers with the age of approximately 55 ± 5 years. All the samples were immediately immersed in 10% formalin followed by further histological processing. Elliptical skin samples were divided across their long axis before embedding them in the paraffin mould. In the mould, they were oriented in such a way that their cut edges were directed as cutting surfaces. Topographic sites where the skin samples were obtained are illustrated in Figure 1.

### 2.1. Sample Collection

- At shoulder joint, the skin samples were obtained slightly lateral to surface projection overlying the acromion process of scapula. Sections taken along the circumferential line of joint were considered as “horizontal,” while perpendicular to it as “vertical.”
- Over the wrist area, skin samples in 2 directions were obtained at the side of proximal crease line of flexor surface.
- At ankle area, skin samples were collected at the site immediately above the topographic site of insertion of tendocalcaneous.
- Forearm skin samples were collected over the middle of flexor compartment of the forearm between midpoint of elbow joint and wrist joint.
- At thigh region, samples were collected along the midpoint on thigh between pubic tubercle and medial condyle of tibia.

### 2.2. Histological Processing and Image Acquisition

Histological sections were stained by special stain Verhoeff-Van Gieson method for the selective demonstration of collagen and elastic fibres [8]. Digital images were acquired at 20x magnification with the standard resolution of 694 × 516 VGA using Progress capture Pro 2.1-Jenoptic microscopic camera fitted to inverted phase contrast camera. From each sample three images were obtained in 3 different microscopic fields.

### 2.3. Image Analysis

**Figure 2:**
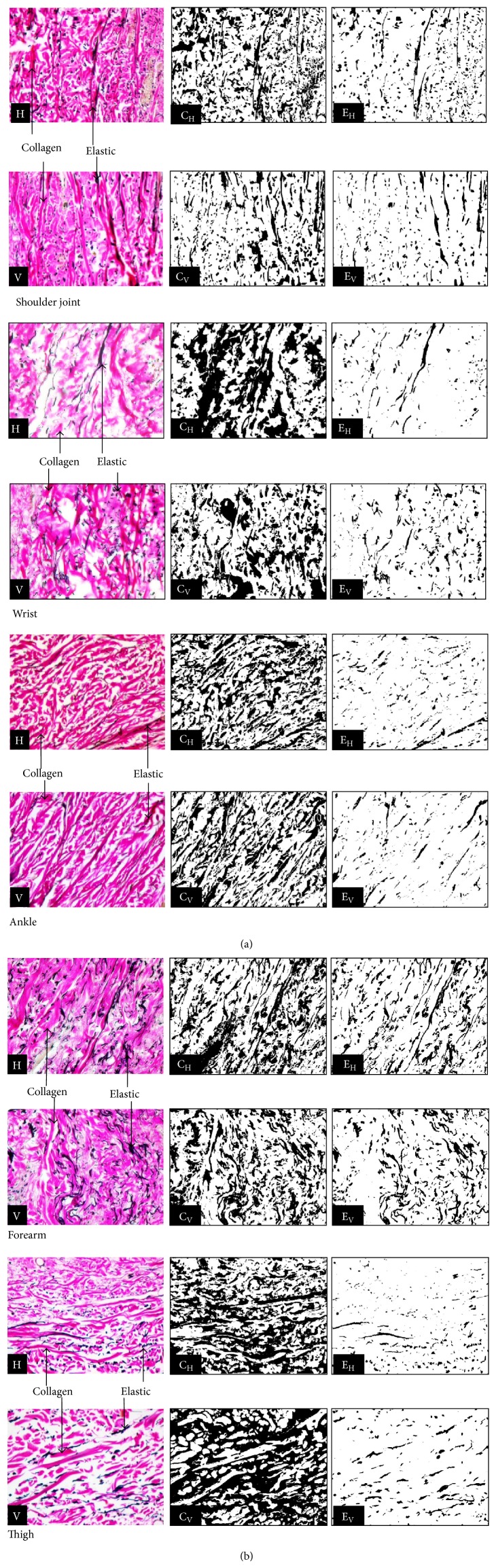
Verhoeff-Van Gieson staining appearance of collagen (pink colour) and elastic fibres (black colour) in horizontal (H) and vertical (V) sections of shoulder joint, wrist, ankle, forearm, and thigh areas. Their pattern of segmentation by tissue-quant software is shown in adjacent photographs. [C_H_: collagen in horizontal, C_V_: collagen in vertical, E_H_: elastic in horizontal, and E_V_: elastic in vertical directions].

Images obtained from special stained slides were subjected to analysis by the software “tissue-quant” version 1.0. This software measures the area occupied by the coloured structure of interest in terms of number of pixels assigned to positively stained area in the image. This measure corresponds to the quantitative fraction of the structure to be analysed. Tissue-quant analysis needs as prerequisites the segmentation of colour and its shades of interest from the rest of the coloured structures (Figure 2). The total number of pixels corresponding to the target area of coloured structure is then converted to percentage value by proper calculation [7].

### 2.4. Analyzed Morphometric Parameters

#### 2.4.1. Quantitative Fraction Analysis

Image analysis results obtained in terms of percentage area occupied by the collagen and elastic fibres were termed as quantitative fraction. Mean value with standard deviation was calculated and the differences of variables between the samples of two orientations were analysed statistically by employing the paired sample* t*-test with 95% confidence interval (CI) using SPSS version 5. *P* < 0.05 is considered to be statistically significant.

#### 2.4.2. Ratio Value Analysis

The collagen and elastic fibre content in horizontally obtained sections were denoted as C_H_ and E_H_, respectively, while for their vertical directions as C_V_ and E_V_, respectively. The ratio between C_H_ and C_V_ was calculated by dividing the C_V_ value by C_H_ and it was denoted by C_V_/C_H_ ratio. Similarly, the ratio between E_H_ and E_V_ was calculated by dividing the E_V_ value by E_H_ and it was denoted by E_V_/E_H_ ratio. These ratios were expressed in “ratio values” which implies proportionate changes in content of “vertical” with respect to its “horizontal” counterpart [1].

## 3. Results

### 3.1. Quantitative Fraction Analysis

The mean quantitative fraction with standard deviation (SD) and mean 95% confidence interval (CI) for collagen in horizontal (C_H_) and collagen in vertical direction (C_V_) with the level of statistical significance (*P* values) between two directions are depicted in Table 1.

The mean C_H_ of dermis overlying shoulder joint was 53.91% and that of C_V_ was 49.03% with a significant difference between two directions (*P* = 0.005). The C_H_ in wrist area was 56.12% and that of its C_V_ was 60.32%. The wide difference between C_H_ and C_V_ confirmed the statistical significance with *P* = 0.016. In the ankle, C_H_ was 61.61% and C_V_ = 62.37%. The difference in the ankle area was very narrow; thus it was statistically insignificant (*P* = 0.640). The C_H_ and C_V_ at forearm were similar to each other, that is, 52.84% and 52.56%, respectively, without any notable difference (*P* = 0.856). Dermis of the thigh area, similar to forearm, also exhibited close ranges of collagen content between horizontal (C_H_ = 52.12%) and vertical (C_V_ = 52.06%) directions. The level of significance in the difference was statistically insignificant (*P* = 0.964).

The mean quantitative fraction with standard deviation (SD) and mean 95% confidence interval (CI) for elastic fibre content between horizontal (E_H_) and vertical (E_V_) directions with its level of statistical significance of differences (*P* values) are depicted in Table 2.

The content of elastic fibres in skin of shoulder joint was 10.34% and 13.07% for E_H_ and E_V_, respectively. The difference was statistically significant (*P* = 0.011). In the wrist, E_H_ was 6.36% and it was much lesser than its E_V_ (8.20%) making remarkable difference which was statistically significant (*P* = 0.014). In the ankle, E_H_ was 5.06% and E_V_ was 8.14% with the statistical significance (*P* = 0.001). Similar to collagen at forearm, the elastic content was also more or less the same, as E_H_ was 14.38% and E_V_ was 14.28% (*P* = 0.892). Unlike the collagen, in the thigh, the amount of elastic showed the significant difference in its content (*P* = 0.028) between horizontal (E_H_ = 14.68%) and vertical (E_V_ = 12.32%) directions.

### 3.2. Ratio Value Analysis

Results of various ratio values of collagen and elastic fibres between horizontal and vertical directions are tabulated in Table 3. For description purpose, the ratio values obtained from statistically significant (*P* < 0.05) quantitative fractions were considered.

## 4. Discussion

A quantitative study of dermal collagen using computerized digital image analysis with comparison of its biochemical analysis was reported to be significantly correlated. The reason for this observation was attributed to the pattern of distribution of collagen irrespective of its type in the dermis [9]. In the human body, the collagen fibres display basket weave like pattern with the random organization [10]. Although the collagen fibre density evaluation normally does not show changes with age, decrease in its content was reported with the observation in individual variations [9]. Even the elastic fibre distribution assay in this aspect was also found to have no variation with respect to sex or age [11].

Stereological analysis of dermal collagen and elastic fibres done by Vitellaro-Zuccarello et al. reported constant volume of collagen throughout the depth of the dermis and increased density of the collagen as age advances in both sexes up to 30–40 years. On the other hand, the elastic fibre's volume and diameter vary in dermal layers and the increment is observed in reticular dermis particularly in males till first decade of life [12]. The elastin content differs and distribution is dependent on the dermal area as it is different among the subjects [13]. In response to stretch, both elastic and collagen fibre's realignment are observed [14].

Subcutaneous or fascial tensile reduction sutures tend to apply negligible tension that in turn plays an effective role in the reduction of recurrence of keloids or hypertrophic scars [15]. The quantitative fraction evaluation of dermal collagen and elastic fibres in the extremities of the body is highly intricate and subjective due to involvement of joints. The present study involves 3 joint areas (shoulder joint, wrist, and ankle) and two other nonstretchable areas (thigh and forearm). For joint area, the evaluation was made under two factors involving burst force exerted over the stretched skin of flexed/adducted joint (e.g., the shoulder joint) and stretch force over skin during movement at joints that are not acutely bent in rest position (e.g., wrist and ankle joint).

### 4.1. Shoulder Joint Area

During normal adducted position at rest, the maximum stretch on the shoulder skin over deltoid region causing burst force with the tendency to create wound in vertical direction. To counteract this force in nature, strength is required along horizontal direction. Thus, the collagen deposition (that provides strength) predominates along the horizontal direction. This was evident from our data in which significant higher collagen fibre content in horizontal direction compared to the vertical direction (C_H_ = 53.91% versus C_V_ = 49.03%, *P* = 0.005) was seen. To compensate between excess stretching and laxity on one or another surface of joint during movement, elastic fibres necessitate increased concentration in vertical direction, that is, perpendicular to the joint line. This was confirmed with our findings in which elastic fibre content (E_V_ = 13.07%) was significantly higher than horizontal (E_H_ = 10.34%) direction (*P* = 0.011).

Thus, when the scar is placed in vertical direction over deltoid region, the burst force during adducted position tends to exhibit wide and/or hypertrophic scar in due course of time. On the other hand, when the scar is placed along horizontal direction, the elastic content in vertical direction is divided and exerts pull on the wound edge producing stretched and/or hypertrophied scar (gaping). Also, the effect of gravity aggravates the force on the horizontal wound edge exerted by divided elastic fibres. Since surgical incision is made along the horizontal direction, the tension produced by elastic fibres on wound/scar edge is probably weaker than burst force due to adducted position (rest position); resulting scar will be better if incision is horizontally placed compared to vertically.

### 4.2. Wrist and Ankle

Similar to shoulder joint area, the functional correlation of elastic fibres in vertical direction is comparable with that of shoulder joint area as the quantitative fraction of elastic fibre was significantly increased at vertical direction at wrist (E_H_ = 6.36 and E_V_ = 8.20 with *P* = 0.014) and at ankle (E_H_ = 5.06 and E_V_ = 8.14 with *P* = 0.001) areas.

However, in terms of collagen content, both wrist and ankle exhibit higher content of collagen in vertical direction which is contrary to shoulder joint area. But the difference was observed to be significant in wrist (C_H_ versus C_V_, *P* = 0.016) and insignificant at ankle (C_H_ versus C_V_, *P* = 0.640). This diverged fact of collagen content may be considered to be anatomical (rest) position of the joint which in turn could be attributed to the facts of burst force versus stretch force as shown in Table 4.

### 4.3. Forearm and Thigh Area

Since there is a minimal effect of movement and gravity over the thigh and forearm skin, no significant differences in the collagen and elastic fibre content between horizontal and vertical directions were to be expected. The results of current study partially support this hypothesis in terms of elastic content except that significant difference in the elastic fibre content over horizontal direction of the thigh region was noted. This becomes obligatory due to a possible stretch force produced by the slow circumferential tissue expansion of bulky thigh with growth of the body that necessitates deposition of more elastic along horizontal direction compared to vertical (E_H_ > E_V_; *P* = 0.028). Contrary to this, the circumferential growth of forearm is less as compared to that of thigh region; the above mentioned observation of thigh is unseen at forearm. As a result, there was no significant difference in elastic content of forearm between two directions (E_H_ = 14.38%, E_V_ = 14.28%, *P* = 0.892).

### 4.4. Ratio Value Evaluation (Table 3)

In the shoulder joint area the C_V_/C_H_ ratio value was less than 1. Horizontal wound edges on this area will often have less distracting force during movements as compared to the burst force on vertical wound edge during rest period. Clinical experience also shows that lower is the value and better is the long term result of horizontally placed scar.

In the wrist area, where the C_V_/C_H_ ratio value was more than 1, burst force produced in horizontal direction during movements (mainly in extension) necessitates providing maximum strength to reduce damage. Due to this, probably collagen content in vertical direction was more.

In the thigh area, where the E_V_/E_H_ ratio value was less than 1, the slow expansion of the tissues causes maximum stretch force in horizontal direction. Therefore elastic content was significantly higher in horizontal direction than in vertical.

In all 3 joint areas (shoulder joint, ankle, and wrist) with the E_V_/E_H_ ratio value more than 1, the constant stretching in vertical direction due to either embryologic growth pattern or gradual growth after birth might have influenced the higher content of elastic fibres along vertical directions (Table 2).

## 5. Conclusion

The analysis of unequal distribution of dermal collagen and elastic fibres in the region of extremities is a complex process and it is solely dependent on the anatomical perspective of the joints involved. The burst force and the stretch force effects at the joint area and corresponding profiles of quantitative fraction of dermal elements need to be taken into consideration before performing surgical incision. In other areas (forearm and thigh) these factors may have negligible effects.

## Figures and Tables

**Table 1:** Descriptive statistics of collagen fibre assay between horizontal (C_H_) and vertical (C_V_) directions from the areas of extremity region of human cadaver.

Area	Horizontal (C_H_)	Vertical (C_V_)	Mean 95% CI	*P* value
	Mean (SD)	Mean (SD)		
Shoulder joint	53.91 (12.3)	49.03 (10.6)	4.88	0.005^*^
Wrist	56.12 (12.0)	60.32 (11.2)	−4.19	0.016^*^
Ankle	61.61 (7.7)	62.37 (8.1)	−0.75	0.640
Forearm	52.84 (12.2)	52.56 (12.1)	0.28	0.856
Thigh	52.12 (12.3)	52.06 (14.1)	0.06	0.964

^*^Indicates statistically significant difference of content between horizontal and vertical direction (*P* < 0.05).

**Table 2:** Descriptive statistics of elastic fibre assay between horizontal (E_H_) and vertical (E_V_) direction from the areas of extremity region of human cadaver.

Area	Horizontal (E_H_)	Vertical (E_V_)	Mean 95% CI	*P* value
	Mean (SD)	Mean (SD)		
Shoulder joint	10.34 (4.5)	13.07 (5.9)	−2.73	0.011^*^
Wrist	6.36 (2.6)	8.20 (3.5)	−1.84	0.014^*^
Ankle	5.06 (2.2)	8.14 (4.2)	−3.07	0.001^*^
Forearm	14.38 (4.7)	14.28 (6.4)	0.10	0.892
Thigh	14.68 (6.7)	12.32 (5.4)	2.36	0.028^*^

^*^Indicates statistically significant difference in the content between horizontal and vertical direction (*P* < 0.05).

**Table 3:** Descriptive analysis of ratio values of collagen (C) and elastic (E) fibres with respect to horizontal (H) and vertical (V) directions.

Areas	C_V_/C_H_ ratio value	E_V_/E_H_ ratio value
Shoulder joint	0.90^#^	1.26^#^
Wrist	1.07^#^	1.28^#^
Ankle	1.01	1.60^#^
Forearm	0.99	0.99
Thigh	0.99	0.83^#^

^#^Ratio values of statistically significant quantitative fraction (*P* < 0.05).

**Table 4:** Force effects at joint areas in anatomical position and during various movements.

Force applicable	Shoulder joint	Wrist area	Ankle area
Burst force due to position	Maximum	Minimum	Moderate
Stretch force due to movement	Minimum	Maximum	Moderate
